# 3D-printed tooth for caries excavation

**DOI:** 10.1186/s12909-024-06230-3

**Published:** 2024-10-31

**Authors:** Lisanne Carnier, Michael del Hougne, Marc Schmitter, Christian Höhne

**Affiliations:** Department of Prosthodontics, University of Würzburg, Pleicherwall 2, 97070 Würzburg, Germany

**Keywords:** Educational Technology, Patient Simulation, Clinical Skills, Printing, Three-dimensional, Prosthodontic, Tooth Preparation, Dental Caries, Dental Pulp Capping, Root Canal Therapy

## Abstract

**Background:**

To date, no suitable model tooth has been available for all standard restorative and prosthodontic procedures. To fill this gap, a realistic multilayer tooth with enamel, dentin, integrated caries, pulp, and electrometric and X-ray imaging abilities was developed. The aim of this study was to test the printed tooth while focusing on caries excavation and pulp capping.

**Methods:**

Based on micro-CT data, a tooth was designed and produced via 3D printing. A total of 396 teeth were tested and evaluated by 66 fourth- and fifth-year students experienced in caries excavation at standard typodonts, extracted teeth and patients. They excavated the caries and capped the pulp on six teeth and rated them in a questionnaire.

**Results:**

Compared with natural teeth, the printed teeth were generally rated positively and significantly better in all criteria than typodonts used previously (*p* < .001). They were rated as a suitable training option (Ø 2.3 ± 0.9) with fair examination conditions (Ø 2.1 ± 0.8) and easy to use (Ø 2.0 ± 0.8). Subjective learning success was also significantly greater (Ø 2.3 ± 0.9) than that of standard typodonts (Ø 3.2 ± 1.1) (*p* < .001). In general, the students desired more exercises with 3D-printed teeth for their studies (Ø 1.8 ± 0.8).

**Conclusions:**

Multilayered 3D-printed teeth were successfully tested and can improve and extend the teaching methods used for caries excavation and pulp capping. Its other abilities will be tested in subsequent studies.

**Year of the Study:**

2023.

## Background

Practical teaching of caries excavation begins in the third year at most German universities with excavation exercises on the phantom head, according to the old and new dental licensing regulations. For these exercises, standard model teeth are used, commonly prepared in advance by the students themselves to simulate carious lesions. Often models from KaVo Dental GmbH (Germany) or frasaco GmbH (Germany) are utilised in dental education. These model teeth consist of a single material. Additionally, previously extracted natural teeth were collected and used for the exercises on the phantom head. The use of these tools for dental teaching has been criticised by many university ethics committees in recent years because of the property of the extracted teeth and the extent to which prior patient consent was necessary for subsequent educational use [1]. Since then, some ethics committees have imposed more stringent requirements for patient information and documentation of consent for university use of teeth. This additional administrative burden could significantly decrease the future willingness of some dentists to collect suitable extracted teeth [2]. Furthermore, their use is controversial from a hygienic point of view, as they represent a potential source of infection. To avoid this risk, thorough disinfection or sterilisation of the teeth is needed, which is associated with increased time and costs. Furthermore, the properties of the natural tooth are altered by these pretreatments, underlining the need for a model specifically designed for training. In addition, it is often impossible for students to obtain teeth in sufficient numbers and under sufficient conditions [3]. This yields uneven conditions for students, increasing difficulties for supervising staff trying to assess consistently and uneven learning outcomes across courses. Some manufacturers offer special model teeth for teaching caries excavation, representing an expensive alternative and thus suitable for teaching only to a limited extent. With respect to former studies [4–10], a tooth capable of several training situations was developed to overcome this issue, including caries excavation, direct and indirect pulp capping, root canal treatment with electrometric measurement of the length and X-ray image abilities, dentin post preparation and core build-ups with different posts and crown preparations.

The aim of the present and first studies with this new multilayer 3D-printed tooth was to evaluate its cost effectiveness and students’ preference when comparing to natural and typodont teeth.

The null hypothesis was that the printed tooth cannot achieve satisfactory acceptance levels and ratings from the students.

The desired learning outcomes were to improve students’ caries excavation and pulp capping abilities.

## Methods

The Institutional Review Board (University of Würzburg, Germany) approved this study type, and a general exemption for the usage of printed teeth in education was granted. This was also confirmed by the use of anonymized existing scans and radiological data.

### Design of the printed tooth

The printable tooth developed for this study was a reconstruction of an extracted left mandibular first molar, which was voluntarily and anonymously provided by the donor for these purposes. Initially, a micro-CT scan of the extracted tooth with a resolution of 2 microns was created for this purpose by the “Fraunhofer Institute for Integrated Circuits” (Fraunhofer IIS, Erlangen, Germany). Based on the created micro-CT data, the printable tooth was reconstructed using 3D Slicer (www.slicer.org) and Geomagic Design X (3D Systems, Rock Hill, USA). The 3D reconstruction of the tooth was divided into three distinct parts: the tooth root, the dentin with pulp chamber, and the enamel. This enabled the printing of three components separately. In the next step, the reconstruction was imported as a wavefront OBJ file into PreForm 3.29.0 (Formlabs Inc., Somerville, Massachusetts, USA) and prepared for printing.

### Production of the teeth

A Form 3B + stereolithography (SLA) desktop 3D printer (Formlabs Inc.) was utilised for the additive manufacturing of the tooth. As the hard substances on natural teeth differ in both colour and hardness, different modified resins were utilised for printing. For replication of dentin parts Model Resin V3 (RS-F2-DMBE-03, Formlabs Inc.) and for enamel parts Rigid 4000 Resin (RS-F2-RGWH-01, Formlabs Inc.) was utilised. The postprinting process was conducted in accordance with the manufacturer’s instructions. Printed items were processed in Form Wash & Cure units (Formlabs Inc.), washed for 5 min with 100% isopropyl alcohol (VWR International, Radnor, Pennsylvania, USA), separated from the platform and finally washed in a rinsing station (Form 3 Finish Kit, FK-F3-01, Formlabs Inc.) with isopropyl alcohol again. Subsequently, the tooth components were washed for another 4 min in two different ultrasonic baths (Sonorex, Bandelin electronic GmbH & Co. KG, Berlin, Germany) supplemented with isopropyl alcohol. Afterwards, all the items were air-dried.

### Completion of the teeth after printing

**Fig. 1:**
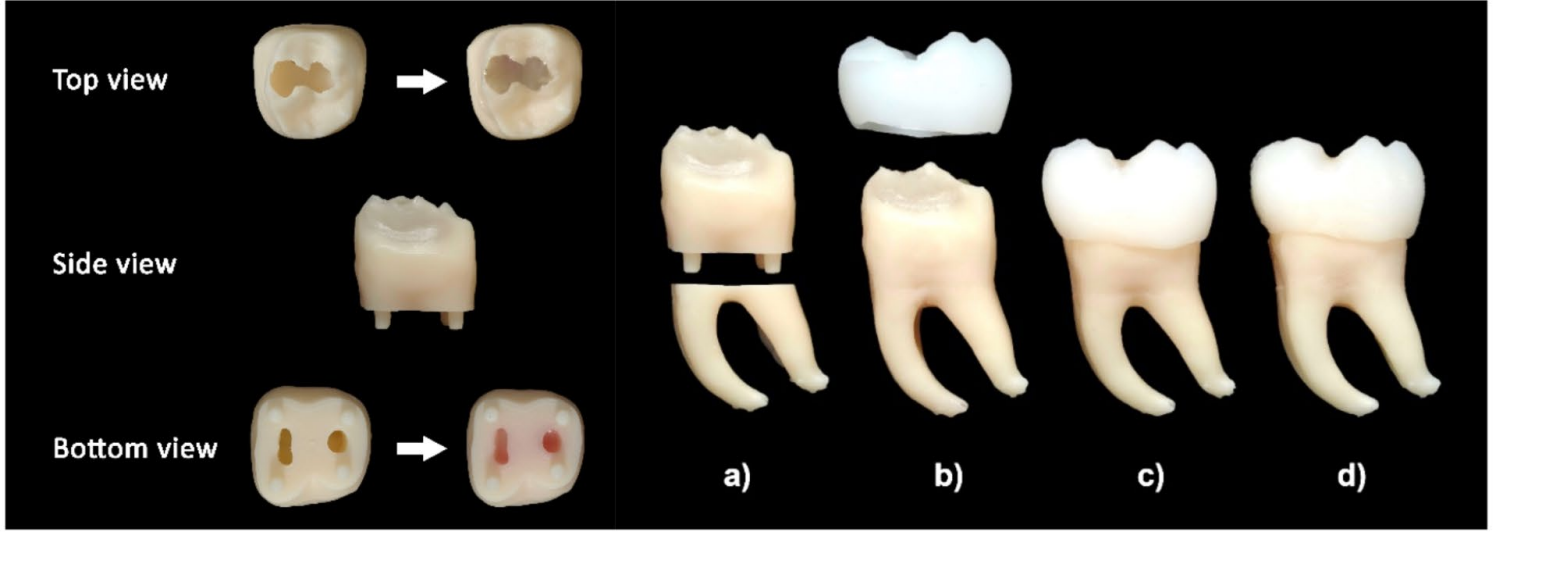
Preparation of coronal dentine segments from different views (coronal, apical and side views). Coronal view: coronal dentine before and after application of the caries material Bottom view: coronal dentine before and after application of the pulp material. Manual assembly of the individual parts: **(a)** prepared coronal and root dentin before bonding; **(b)** combined dentine part before bonding with the enamel layer; **(c)** finished bonded tooth before varnishing the enamel layer; **(d)** finished tooth after curing the enamel layer

To replicate pulp and caries, coronal dentin was restored with different optimised flexible photopolymers (Fig. 1). In the first step, the coronal dentin was restored from below with a material that replicates the pulp (Fig. 1, apical view), followed by curing in a light polymerisation unit (HiLite^®^ power, Heraeus Holding GmbH, Hanau, Germany). The opening at the top of the same component was restored with material representing artificial caries (Fig. 1, coronal view). The artificial caries was also light-cured. The coronal dentin from Fig. 1 was bonded to the root dentin (Fig. 1a, b). The teeth were again exposed to light to cure the adhesive bond, followed by full postcuring for 15 min at 60 °C in the Form Curing Unit (Formlabs, Inc.). After this, the enamel layer was assembled on the dentin (Fig. 1c), and the bonded joint was cured. A thin layer of light-curing one-component varnish was applied and cured to give the artificial enamel surface a more natural appearance.

### Training on the modular model

**Fig. 2:**
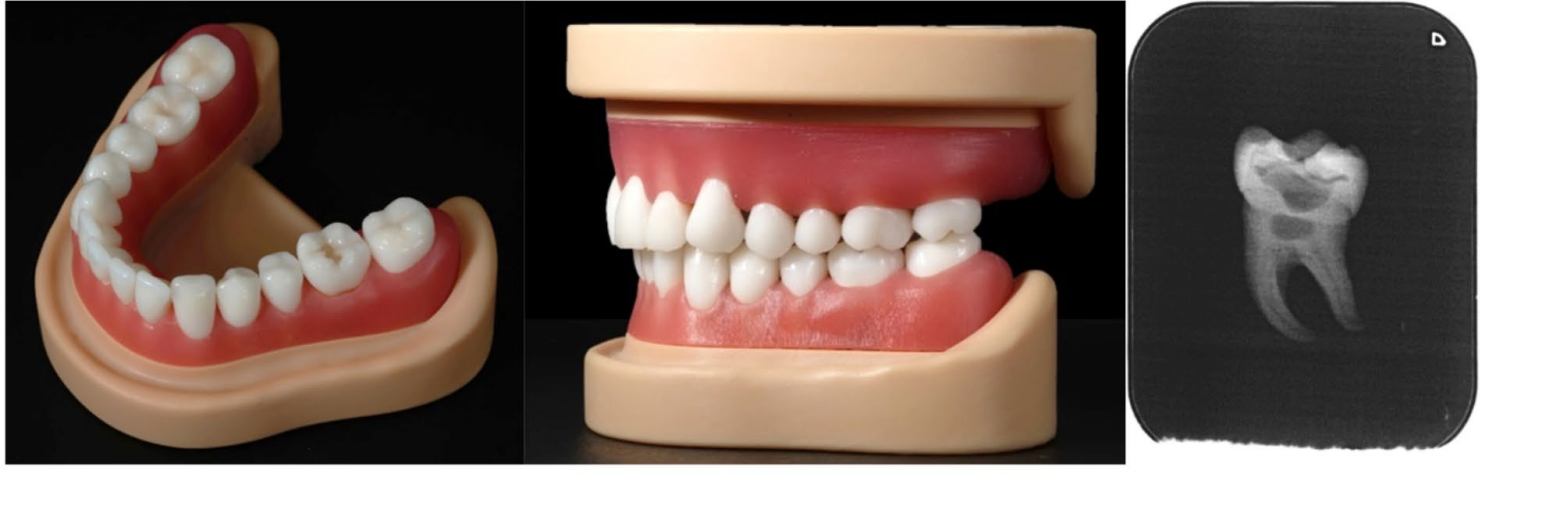
Printed tooth 36 inserted in the mandibular model (left) and in occlusion with the maxillary model (middle). Real radiograph of the printed molar (right)

**Fig. 3:**
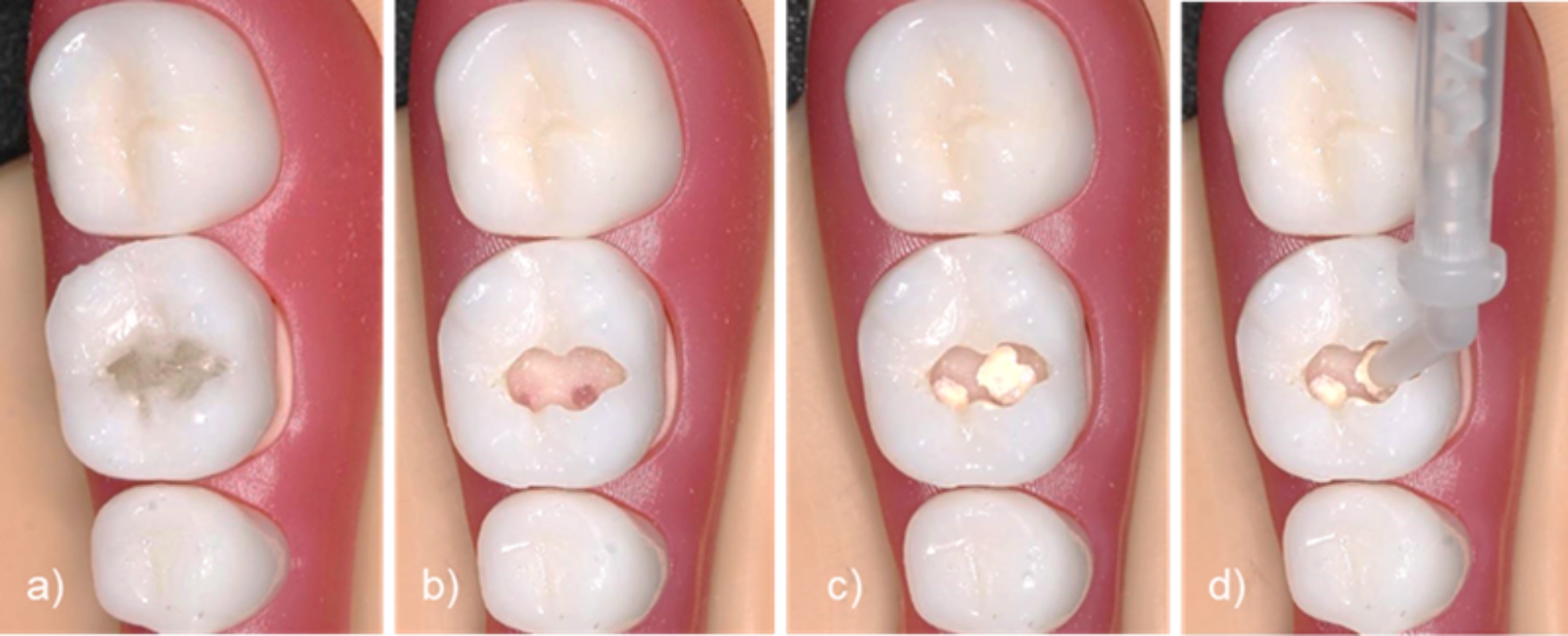
Representation of the exercise sequence: **(a)** printed tooth inserted into the model; **(b)** caries excavation with iatrogenic pulp exposure (optional); **(c)** pulp capping with calcium hydroxide paste; **(d)** build-up restoration of the cavity

A total of 66 fourth- and fifth-year students (43 women and 23 men, between 21 and 37 years of age with an average age of 25.4 years) participated in a voluntary three-day hands-on course. They had already successfully completed their first clinical treatment course in conservative dentistry. All students had worked with natural tooth models and standard model teeth during their studies. They were familiar with current teaching methods in the field of caries excavation and pulp capping. Each student received six printed molars and one matching 3D-printed maxillary and mandibular modular model (Fig. 2, left and middle). In addition, the students were given an actual radiograph of the printed molar for better orientation (Fig. 2, right). The students excavated the caries sections of the printed teeth using carbide burs - no specific sequence of burs was predetermined. After excavation, the 3D-printed teeth were capped with calcium hydroxide paste and restored with composite. Pulp capping was implemented during the exercise to train iatrogenic pulp exposure. Figure 3 provides an overview of the training procedure.

### After-practice questionnaire

**Fig. 4:**
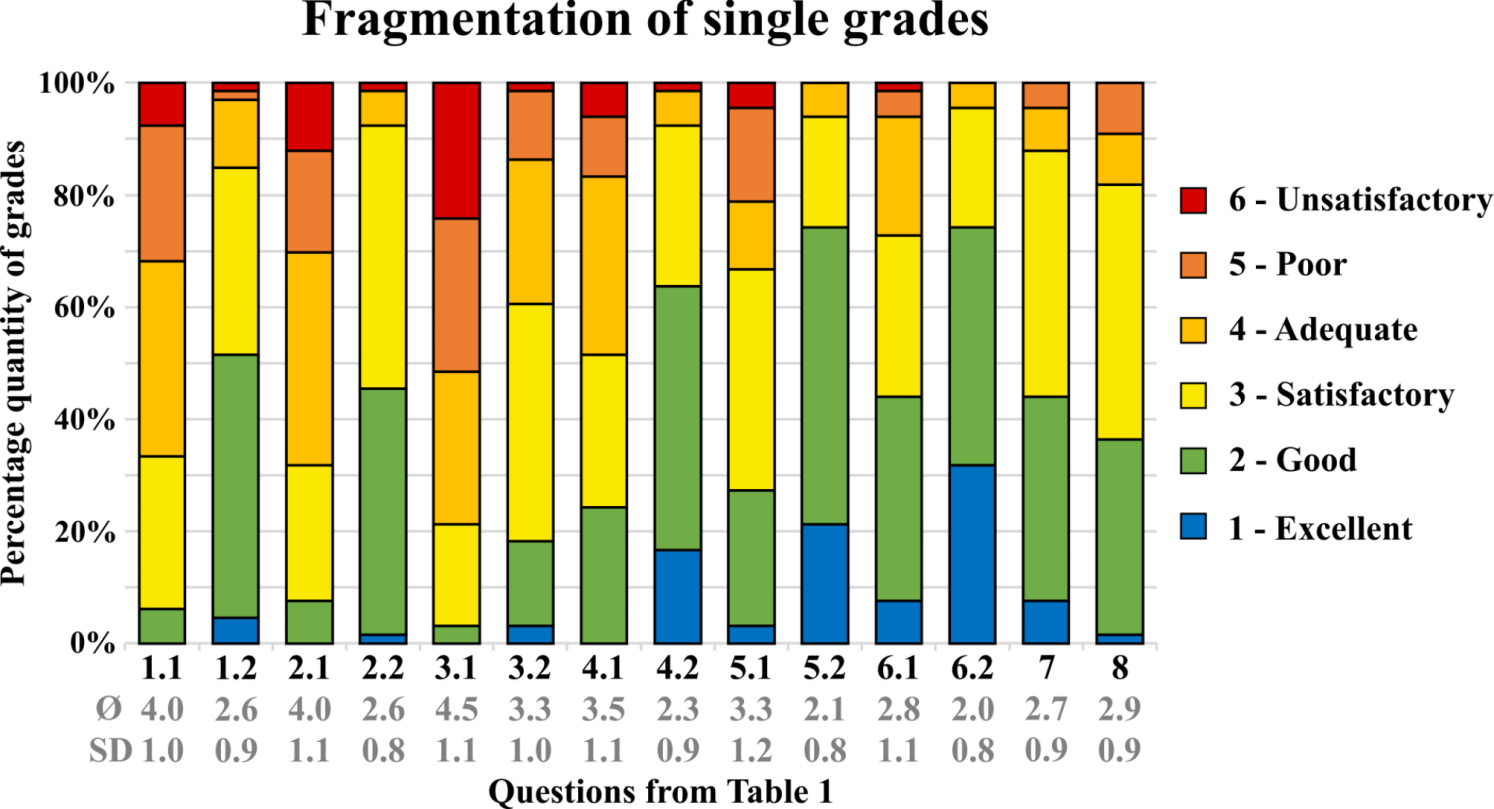
Results from the questionnaire for items 1.1 to 8. The percentages of given grades are displayed in this bar chart

**Fig. 5:**
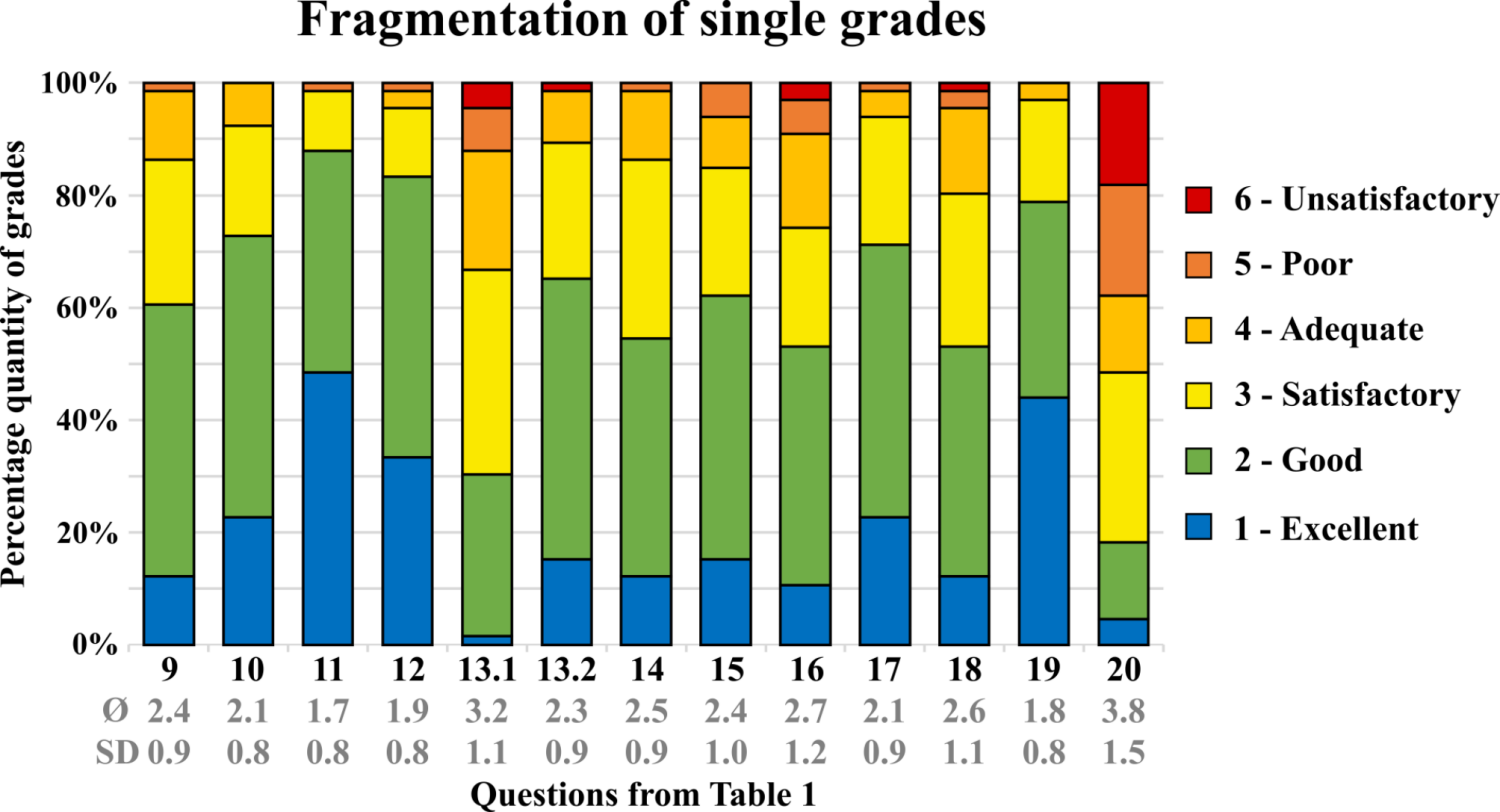
Results from the questionnaire for items 9 to 20. The percentages of given grades are displayed in this bar chart

To evaluate the suitability of the printed tooth for use in dental teaching, the students answered an online questionnaire (Table 1) after completion of the hands-on training. This was created with the EvaSys evaluation system (Electric Paper Evaluationssysteme GmbH, Lüneburg, Germany) and assisted by the “Institute for Medical Teaching and Medical Educational Research” of the University of Würzburg. The questionnaire was administered in German, and the results were subsequently translated. The aim of this study was to compare the new tooth to natural teeth and standard typodont teeth. Similar questionnaires have already been used for student evaluation in other studies with printed teeth. However, the questions were adapted to the present study. The evaluation was based on German school grades and closed-format rating scale questions (1 = Excellent, 2 = Good, 3 = Satisfactory, 4 = Adequate, 5 = Poor, 6 = Unsatisfactory). For visualisation, the mean grade and standard deviation were calculated and are displayed in bar charts (Figs. 4 and 5). The distributions of the grades are shown as percentages. For the reliability of the questionnaire, Cronbach’s alpha was calculated. Significant differences between groups were calculated using the Mann‒Whitney U test with the statistical program SPSS (SPSS 29, IBM Corp., NY, USA). Values under *p* = .05 were considered significantly different. The questionnaire ended with two free text questions. The students were asked to evaluate the possible disadvantages and advantages of the printed tooth. Evaluation of the free text questions was handled by two independent researchers to group and count similar answers.

**Table 1:** The questionnaire for the evaluation of the printed tooth

**Comparison of the model teeth to natural teeth**
1. realistic feeling of the teeth during caries excavation 1.1. KaVo/Frasaco 1.2 printed tooth 2. realistic consistency of the caries lesion 2.1. KaVo/Frasaco 2.2 printed tooth 3. realistic appearance of the caries lesion 3.1. KaVo/Frasaco 3.2 printed tooth 4. appropriate exercise option 4.1. KaVo/Frasaco 4.2 printed tooth 5. fair examination conditions 5.1. KaVo/Frasaco 5.2 printed tooth 6. easy to use 6.1. KaVo/Frasaco 6.2 printed tooth
**Comparison of the printed teeth to natural teeth**
7. realistic color differentiation between enamel and dentin 8. realistic hardness differentiation between enamel and dentin 9. realistic core build-ups 10. realistic pulp capping 11. realistic shape of the printed tooth 12. realistic X-ray
**Assessment of the learning outcome**
**My subjective learning success was greatest …** 13.1 … with KaVo/Frasaco teeth. 13.2 … with the printed teeth. **After the hands-on course**,** I feel well prepared …** 14. … in caries excavation. 15. … in pulp cappings. 16. … in core build-ups.
**Assessment of the learning process**
17. The printed teeth raised my enthusiasm to improve my skills in caries excavation. 18. The printed teeth helped me improve my skills in caries excavation. 19. I am interested in more exercises with printed teeth for my studies. 20. I could imagine the entire training in preparation for treating patients with 3D printed teeth rather than natural ones.
**Free text questions**
21. What could be improved about the printed teeth? 22. What are the advantages of the printed teeth?

## Results

The internal consistency of the questionnaire was good, with a Cronbach’s alpha of 0.86. The results of the questionnaire (Table 1) are shown in Figs. 4 and 5.

### Production and completion of the tooth

In preparation for the hands-on course, a total of 396 printed teeth were fabricated, with a total printing time of 62 h and 58 min. The printing time for all components for one tooth was therefore approximately 10 min. With a total washing time of 119 min and a total assembly and finishing time of 2 min per tooth, the total fabrication time was 4689 min, which corresponds to approximately 78 h. The pure material costs were 0.18€ per printed tooth and, together with the salary of a dental technician for the completion 0.85€. In addition, special equipment (3D + printer Form 3B, Form Cure and Wash, PreForm software) of approximately €8.500 was needed.

### Comparison to a standard model tooth

The printed teeth and the typodont teeth used previously were evaluated for comparison with natural teeth. The printed teeth were rated significantly better than the typodont teeth used previously in all categories. The degree of realism of the sensation when excavating the printed tooth was graded as Ø 2.6 ± 0.9 (Fig. 4 1.2), whereas that of the standard typodont tooth was graded as Ø 4.0 ± 1.0 (Fig. 4 1.1, *p* < .001). The consistency of the caries of the printed tooth (Fig. 4 2.2: Ø 2.6 ± 0.8) and its appearance (Fig. 4 2.1: Ø 3.3 ± 1.1) were also rated as significantly closer to reality than the standard typodont teeth. In addition, the printed tooth offers significantly fairer examination conditions (Fig. 4 5.2: Ø 2.1 ± 0.8) and is significantly easier to handle (Fig. 4 6.2: Ø 2.0 ± 0.8) (all *p* < .001).

### Comparison to a natural tooth

The realism of the printed tooth was evaluated in comparison with natural teeth according to various criteria. The printed teeth obtained an average rating of good across all categories. The color difference between the enamel and dentin layers was Ø 2.7 ± 0.9 (Fig. 4.7), and the hardness difference was Ø 2.9 ± 0.9 (Fig. 4.8). The realism when placing build-up restorations was rated as good (Fig. 5.9: Ø 2.4 ± 0.9), as was the capping of the pulp of the printed tooth (Fig. 5.10: Ø 2.1 ± 0.8). The shape of the printed tooth was graded to Ø 1.7 ± 0.8 (Fig. 5.11), and its radiograph with Ø 1.9 ± 0.8 (Fig. 5.12).

### Assessment of the learning outcome

The subjective learning success of the students was significantly better with the printed teeth (Fig. 5 13.2: Ø 2.3 ± 0.9) than with the standard typodont teeth used previously (Fig. 5 13.1: Ø 3.2 ± 1.1, *p* < .001). As a result of the hands-on course, the students felt well prepared for pulp capping (Fig. 5.15: Ø 2.4 ± 1.0) and satisfactorily prepared for the excavation of caries (Fig. 5.14: Ø 2.5 ± 0.9) as well as for the placement of build-up restorations (Fig. 5.16: Ø 2.7 ± 1.2).

### Assessment of the learning process

The printed teeth increased enthusiasm for improving caries excavation skills (Fig. 5.17: Ø 2.1 ± 0.9). With the aid of the printed tooth, the students were able to improve satisfactorily in excavating caries (Fig. 5.18: Ø 2.6 ± 1.1). For their studies, they desired more exercise with printed teeth (Fig. 5.19: Ø 1.8 ± 0.8). Nevertheless, they wanted their entire training in preparation for patient treatment not only with printed teeth but also with natural teeth (Fig. 5.20: Ø 3.8 ± 1.5).

### Free text questions

The results of the free-text questions of the 66 students were analysed. Similar answers were grouped and counted. Both questions were answered by more than 90% of the students.

### Disadvantages of the printed teeth

- bond between caries and the dentin layer too weak (*n* = 16) / too strong (*n* = 1).
- caries material too firm (*n* = 9), too sticky (*n* = 5), or too soft (*n* = 1).
- the enamel layer (*n* = 9) and dentin layer (*n* = 5) are too soft.
- caries is too close to the pulp (*n* = 8).
- the colour contrast between the caries and dentin layers was too weak (*n* = 8).
- retention of the tooth in the model too weak (*n* = 7).
- no change to the teeth necessary (*n* = 7).
- roots and crown fracture too easily (*n* = 5).
- the color of the caries is too unrealistic (*n* = 5).
- opening of the occlusal surface too small (*n* = 4).
- caries consistency not good (*n* = 2).
- transition between the layers too clearly recognizable (*n* = 1).

### Advantages of the printed tooth for education

- more realistic practice opportunity than the typodont teeth used previously (*n* = 21).
- good and realistic exercise for patient treatment (*n* = 11).
- practicing caries excavation (*n* = 3) and pulp capping (*n* = 8) is enabled ahead of treating patients.
- financial relief (*n* = 8) and thus possibilities to practice independent of income (*n* = 2).
- fair and comparable examination conditions (*n* = 8).
- realistic excavation feeling (*n* = 7).
- presence of a pulp (*n* = 6), realistic appearance of the pulp (*n* = 2).
- time savings in preparation of the exercise (*n* = 6).
- more variability of teeth due to additive manufacturing possible (*n* = 5).
- multilayer build-up (*n* = 4).
- more realistic anatomical conditions (*n* = 4).
- extended practice options (*n* = 3).
- realistic caries extension (*n* = 2).
- realistic radiograph (*n* = 2).
- learning effect is greater since caries extension is unknown in advance (*n* = 1).
- easy handling (*n* = 1).

## Discussion

The results of the questionnaire emphasise the potential of the printed tooth and confirm the added value for dental education. In particular, the free-text questions at the end of the questionnaire indicated that students generally welcomed the concept of printed teeth in education. Nevertheless, several suggestions for improvement were expressed, encouraging future modifications of the printed tooth. Frequently mentioned (*n* = 16) was a weak layer bond between the dentin layer and the caries, which could be optimised in the future by pretreatment with other primers. The aforementioned procedures have been successfully used in other applications to increase adhesion [11–13]. The excessive hardness of the caries material (*n* = 9) could be improved in the future by reducing the exposure time during curing or using a softer material. On the other hand, the hardness of the enamel layer (*n* = 9) and the dentin layer (*n* = 5) could be increased by using ceramic-filled special resins; however, the manufacturing costs increase due to significantly higher material prices, and thus, these materials are less suitable for educational use. In addition, it is not certain whether an acceptable hardness of the printed teeth can be achieved with these resins. Since human enamel with a hardness of 350 KHN [14, 15] represents the hardest tissue of the human organism [16], no resin is available on the market yet that can adequately replicate its hardness. Other points of criticism, such as a weak color contrast between the enamel and dentin layers (*n* = 8) or the position of the caries in relation to the pulp (*n* = 8), can be varied and adjusted with little effort. Weak retention of the tooth in the model was also noted (*n* = 7). Increasing retention while maintaining the realistic appearance of the printed tooth could be achieved by encasing the roots of the printed tooth with transparent plastic, increasing the possibility of retention by screwing.

The students also mentioned numerous advantages of the printed tooth. Provision of a more realistic practice option compared to the typodont teeth used thus far (*n* = 21) was stated most frequently. As a result, realistic preparation for patient treatment (*n* = 11) is enabled. The realistic anatomical conditions (*n* = 4), the presence of a pulp (*n* = 6), and the multilayer design (*n* = 4) were also praised, allowing students extended practice opportunities (*n* = 3), such as caries excavation (*n* = 3) and pulp capping (*n* = 8). The students also reported that the sensation of excavating caries was realistic (*n* = 7).

In addition, using the printed tooth eliminates the preparation time for collecting and cleaning natural teeth or placing artificial carious lesions in standard model teeth, which would result in time savings (*n* = 6). The preparation time saved by the printed tooth could provide more practice time in preclinical courses in the future, which in return would lead to an increase in patient safety in clinical treatment courses. In addition, printed teeth provide fair and comparable examination conditions (*n* = 8), facilitating assessment for the supervising staff and providing more consistent learning outcomes in the courses. It is particularly difficult for inexperienced students to determine the therapeutic endpoint during excavation, underscoring the need to develop further training solutions for education in this area. For this purpose, various computer-assisted dental simulators capable of simulating realistic practice scenarios based on virtual reality technology have been developed in recent years. Although these devices can simulate numerous dental procedures, such as caries excavation, crown preparation or the placement of implants, they are rarely utilised in teaching due to their high cost [17, 18]. The financial relief (*n* = 8) provided by printed teeth and thus more income-independent practice opportunities (*n* = 2) were mentioned as advantages. This is due to the fact, that students of the University of Wuerzburg generally purchase their typodont teeth for practices - however, this may differ at other universities and countries. The production cost of the printed tooth is approximately 0.85€ and can even be reduced by the completion of the printed teeth by the students themselves. In addition, the one-time acquisition costs for 3D printing equipment must be considered, although 3D printing equipment is already available at some universities not only for student training but also for patient treatment. The production of the printed teeth could be carried out by the students themselves or by student assistants after a short training period. The price of the single material typodont teeth used previously was approximately two times greater. Thus, the use of the printed tooth results in financial relief for the students. Several other studies addressing the development of printed teeth for dental teaching have reached similar conclusions [6, 8, 9]. Additive manufacturing allows greater variability in tooth morphology, the location of the pulp, caries and their extent (*n* = 5). This approach is advantageous since it is often impossible to obtain suitable teeth in sufficient numbers from all quadrants using natural teeth. Nevertheless, the fabrication of the printed teeth cannot be automated completely yet, requiring some manual steps, as the SLA printer Form 3B+ (Formlabs Inc.) used cannot print multiple materials simultaneously. Stereolithography is an established technique with high precision and excellent surface quality [19]. Some studies have investigated the precision of the Form 2, 3B/3B + SLA printer and emphasised its suitability for use in dentistry [20–22]. The polyjet process is an alternative manufacturing option, allowing simultaneous printing of multiple materials, which has already been used in other studies for the fabrication of printed teeth [23, 24]. Its disadvantage is the significantly higher acquisition costs compared to those of the SLA method. Therefore, this method was not utilised for fabricating the printed teeth for this study. The students’ subjective learning success was significantly greater for printed teeth (Ø 2.3 ± 0.9) than for standard typodont teeth used previously (Ø 3.2 ± 1.1) (*p* < .001). In addition, the printed teeth were rated significantly better than the standard typodont teeth in all categories, allowing the reduction or replacement of their use in the future. However, the students disagreed on entire training in the preparation for patient treatment on only printed teeth instead of natural teeth (Ø 3.8 ± 1.5). Future studies should further investigate this disagreement - however, it could be caused by the stated limitations of the 3D printed teeth. Improvements of the printed tooth could be realised in the future. The psychological component of working with donated human biomaterials is usually considered a privilege by students, aiding professional and personal development. Printed teeth reduce the demand for natural teeth, although they are unable to eliminate the demand and use of natural teeth entirely. Considering the increased requirements of individual ethics committees and difficulties in obtaining extracted natural teeth, these results are particularly useful.

Overall, the multilayer-printed tooth provided adequate opportunities for educational purposes and students attested satisfactory ratings. Thus, the null hypothesis could be rejected. The tooth in this experiment was reconstructed from an extracted left mandibular first molar; however, the findings can be generalised for analogue replications of this method. The manufacturing process could be transferred and adopted for other 3D printer models and materials. Furthermore, the model could be tested and evaluated by experienced professionals to provide valuable feedback in future studies. Although the results are only based on self-reported data, they are encouraging for further investigations and enhancements of the model.

## Conclusions

The suitability of multilayer-printed teeth with integrated caries and pulp for use in dental education was evaluated successfully. Students had the opportunity to practice correct caries excavation and pulp capping on the phantom head with the aid of the printed tooth. The results of the questionnaire showed that the students favoured the use of printed teeth over typodont teeth in education. The printed teeth were rated significantly better in all categories than the standard typodont teeth used previously. On average, the printed tooth was rated as good. As a result, the printed tooth enables fair and comparable conditions in examination situations. In addition, students can be relieved financially due to low manufacturing prices. The students’ subjective learning success was significantly greater with the printed teeth than with the typodont teeth used previously.

## Data Availability

No datasets were generated or analysed during the current study.
